# Safety and effects of transcranial direct current stimulation on hand function in preschool children with hemiplegic cerebral palsy: A pilot study

**DOI:** 10.3389/fnbeh.2022.925122

**Published:** 2022-09-09

**Authors:** Wenjie He, Yuan Huang, Lu He, Liru Liu, Peishan Zeng, Huiying Qiu, Xiaoyue Wang, Hongyu Zhou, Zhaofang Chen, Yi Xu, Jingyi Zhao, Wenda Wang, Hongmei Tang, Kaishou Xu

**Affiliations:** ^1^Department of Rehabilitation, Guangzhou Women and Children's Medical Center, Guangzhou Medical University, Guangzhou, China; ^2^School of Medicine, South China University of Technology, Guangzhou, China

**Keywords:** hemiplegic cerebral palsy, transcranial direct current stimulation, safety, hand function, preschool children

## Abstract

Transcranial direct current stimulation (tDCS) has shown a promising prospect in improving function and spasticity in school-aged children with cerebral palsy, but little is known in preschool children. The aim of this study was to explore the safety and effects of tDCS on hand function in preschool children (aged 3–6 years) with hemiplegic cerebral palsy (HCP). We designed a crossover, single-blind, sham-controlled study in 30 preschool children with HCP, who were recruited to receive one session of sham and one session of active anodal tDCS (1.5 mA, 20 min) on the primary motor cortex of the affected hemisphere, with a 24-h interval between the two sessions. Questionnaire was completed by each participant and their attendants immediately, 90 min, and 24 h after each session to monitor common adverse events of tDCS, such as skin irritation, skin erythema, burning sensation, headache, dizziness, etc. Box and Block Test, Selective Control of the Upper Extremity Scale, Modified Ashworth Scale, and Melbourne Assessment 2 were conducted at baseline, immediately, and 90 min after each session. No severe adverse event occurred during the study and only a few of them felt transient and slight discomfort. Results also showed that all participants performed better at Box and Block Test of the hemiplegic hand immediately after a single anodal tDCS (*P* < 0.05) and this improvement lasted at least 90 min and more than 24 h. However, there was no significant improvement in Selective Control of the Upper Extremity Scale of both hands, Box and Block Test of the non-hemiplegic hand, Modified Ashworth Scale, and Melbourne Assessment 2 of the hemiplegic upper limb (*P* > 0.05). Shortly, this study supported the safety and effects of a single anodal tDCS on improving the manual dexterity of the hemiplegic hand for preschool children with HCP. Further researches with larger samples about the optimal dose and treatment cycle of tDCS for preschool children with HCP are warranted. This study gained the approval of ethics committee of the organization and was registered at chictr.org (ChiCTR2000031141).

## Introduction

Cerebral palsy is characterized as movement and posture disorders with complicated etiology and stable prevalence of 2–3.5 cases per 1,000 live births (Colver et al., 2014; Li et al., 2022). Hemiplegic cerebral palsy (HCP) is the most common type of cerebral palsy, accounting for 44% (Zelnik et al., 2016). Hemiplegic cerebral palsy affects one side of the body and the upper limb is more involved, which causes unimanual dysfunction, impaired dexterity, and poor bimanual coordination, exerting serious negative effects on daily activities throughout their lifetime.

Several types of interventions have been successfully employed to improve hand function for children with HCP in recent years, such as constraint-induced movement therapy and bimanual intensive therapy, which aim at improving motor function by specific upper limb tasks and may facilitate the brain plasticity in a way from the periphery to the center (Gordon et al., 2011; Novak et al., 2020). Meanwhile, the effect was still unsatisfactory for some children and there were a few new techniques, such as transcranial magnetic stimulation and transcranial direct current stimulation (tDCS), combined with intensive therapy to enhance the effect (Duarte Nde et al., 2014; Wu et al., 2022). Transcranial direct current stimulation, a simple and portable non-invasive brain stimulation which works by means of delivering low-level direct current to facilitate or inhibit cortical spontaneous neuronal activity (DaSilva et al., 2011; Brunoni et al., 2012; Marquez et al., 2015), has attracted more and more attention in healthy humans and clinical populations (Shin et al., 2015; Lefaucheur et al., 2017; O'Leary et al., 2021). In healthy volunteers, interesting findings that tDCS could safely enhance memory, emotional regulation, language, attention, and learning processes have been reported (Shin et al., 2015; Ciechanski and Kirton, 2017). In clinical studies, previous findings demonstrated that anodal tDCS was effective for limb-kinetic apraxia in Parkinson's disease, for motor function in stroke patients, for control functions in children with attention deficit/hyperactivity disorder and for symptom reduction in autism spectrum disorder (Kang et al., 2016; Osorio and Brunoni, 2019; Nejati et al., 2020; Park et al., 2022).

For children with HCP, the majority of tDCS researches focused on improving spasticity and lower limb function and only a few researches investigated the tDCS effects on hand function in HCP (Fleming et al., 2018; O'Leary et al., 2021). Most studies showed the improvement in spasticity, gait velocity and cadence, body sway velocity and balance after single or continuous anodal tDCS combined with other therapy (Collange Grecco et al., 2015; Auvichayapat et al., 2017; Grecco et al., 2017). But two studies showed no significant effect in hand function after serial sessions of cathodal tDCS over the contralesional primary motor cortex (Kirton et al., 2017; Gillick et al., 2018). Meanwhile, anodal tDCS, usually applied separately or combined with other traditional therapies, unilaterally over the primary motor cortex (M1) of the affected or more affected hemisphere, safely improved hand function for school-aged children with HCP without serious adverse event reported (Auvichayapat et al., 2017; Moura et al., 2017; Inguaggiato et al., 2019). All of the above researches were primarily conducted in school-aged children and young adults with HCP and there was a paucity of researches about the safety and effects of tDCS on preschool children (aged 3–6 years old). However, preschool children are in a developing stage of cortical excitability and corticospinal excitability (Säisänen et al., 2018). Given the potential mechanism of tDCS, this period might be more critical for its application and rehabilitation of hand function.

The evidence about optimal tDCS current and duration for HCP is still insufficient but some studies have shown that the safety and effects of tDCS could be influenced by density (Krishnan et al., 2015). Current intensities in most studies about tDCS in pediatric populations have ranged from 0.3 to 2.0 mA and the most frequently used intensity in HCP was 1 mA with a duration of 20 min (Krishnan et al., 2015; O'Leary et al., 2021). Notably, relevant researches have indicated that low current (0.7 mA) was too weak to produce measurable corticospinal excitability changes and behavioral effects for individuals with HCP (Gillick et al., 2018; Nemanich et al., 2019). Another pilot study first explored the safety and effects of anodal tDCS at 1.5 mA for 20 min in school-aged children with HCP, whose parameters were on the basis of evidence from stroke in adults (Inguaggiato et al., 2019). The safety and effects of these tDCS parameters (1.5 mA, 20 min) remain unknown in preschool children with HCP.

To fill this gap, we designed this study to investigate the safety and effects of a single anodal tDCS (1.5 mA, 20 min) over the M1 on hand function in preschool children with HCP.

## Methods

Our study was a crossover, single-blind, sham controlled trial, which gained the approval of ethics committee of the organization and was registered at chictr.org (ChiCTR2000031141). All legal guardians of participants signed the informed consent before enrollment.

### Participants

**Figure 1:**
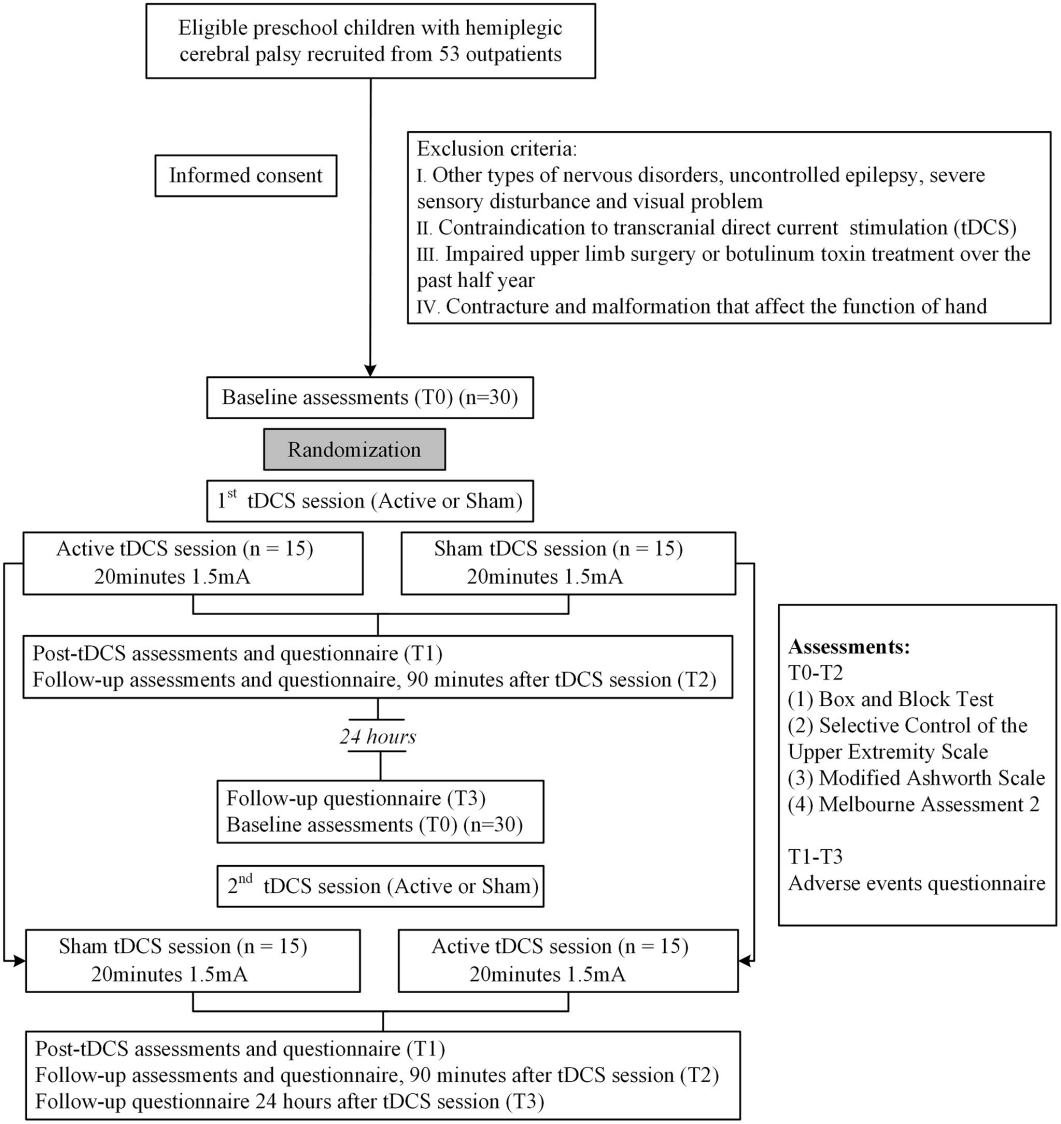
Consort diagram and study flow.

Thirty participants were recruited in the rehabilitation department of Guangzhou Women and Children's Medical Center from September 2019 to February 2020. We screened children (3–6 years old) diagnosed as HCP according to published criteria (Rosenbaum et al., 2007) and categorized as Manual Ability Classification System or Mini-Manual Ability Classification System levels I to II. The exclusion criteria were as follows: (i) other severe illness such as congenital heart disease, uncontrolled epilepsy, leukemia, severe sensory disturbance, and visual problem; (ii) contraindications for tDCS including children with metal or electronic implants, with local skin injury or inflammation, with significantly increased intracranial pressure, with hyperalgesia in the stimulated area, with convulsions or uncontrolled seizure and those who suffered from serious adverse events after tDCS (Antal et al., 2017); (iii) previous botulinum toxin treatment over the past 6 months or preparation for receiving botulinum toxin treatment during trial; (iv) previous surgery of the impaired upper limb. Thirty children completed the entire study. The flow chart of this study was shown in Figure 1.

### Design

All recruited children were randomized into two groups in a 1:1 ratio using a random number table produced by Statistical Product and Service Solutions for Windows (release 26.0, SPSS), and each group received a single session of active anodal tDCS or a single sham tDCS over M1 first and the stimulation was switched after 24 h (crossover phase). Participants and guardians were blind to tDCS assignment. Safety questionnaire was completed immediately (T1), 90 min (T2), and 24 h after tDCS (T3). Assessments of hand function were performed by two independent and occupational therapists at baseline (T0), immediately (T1), and 90 min (T2) after each session. The device was produced by Wuhan Yimai Medical Technology Co., Ltd. and the model was EM8060.

### Interventions

Two 5.5 × 4.0 cm electrodes were placed on the scalp with the anode positioned in the region over the M1 of the affected or more affected hemisphere according to the 10–20 electroencephalogram system, with the cathode electrode placed over the contralateral supraorbital area. The rationale of unilateral stimulation was based on a concept that stimulating the injured brain could enhance motor learning. During active anodal tDCS, a constant current of 1.5 mA was applied for 20 min (with 30 s for ramping up at the beginning and down at the end). The same stimulation protocol was applied in sham tDCS but the current lasted only 30 s. This protocol was proposed by previous tDCS investigation on HCP (Inguaggiato et al., 2019).

## Outcome assessments

### Safety

All participants and their attendants completed an adverse events questionnaire at T1, T2, T3. The questionnaire consisted of eight commonly-reported adverse events (i.e., dizziness, headache, scalp pain, burning sensation, tingling, drowsiness, itching, skin redness) as well as an “other” category that allowed them to describe uncovered experiences/sensations (Brunoni et al., 2011; Krishnan et al., 2015; Reckow et al., 2018). The intensity of adverse events was rated verbally by one of the occupational therapists (i.e., 0, absent; 1, mild; 2, moderate; 3, severe).

### Hand function

The gross motor function of all children was measured by Gross Motor Function Classification System (GMFCS) (Paulson and Vargus-Adams, 2017). The manual abilities were classified by the Manual Ability Classification System (for children over 4 years old) or Mini-Manual Ability Classification System (for children aged 3–4 years old) (Eliasson et al., 2017; Paulson and Vargus-Adams, 2017).

All assessments of hand function were based on the dimensions of international classification of functioning, disability, and health (ICF) (Cieza et al., 2019; Madden and Bundy, 2019) and conducted at T0, T1, and T2. Box and Block Test was used to measure the gross manual dexterity for adults with upper limb paresis and also to determine therapeutic efficiency for children with HCP in clinical rehabilitation, which features advantages of simplicity of operator, reliability, and repeatable measurement (Platz et al., 2005; Jongbloed-Pereboom et al., 2013; Araneda et al., 2019). Melbourne Assessment 2 mainly assessed the movement quality of the upper limb for children with neurological impairment aged 2 years and 6 months to 15 years (Randall et al., 2012, 2014; Elvrum et al., 2016). Selective Control of the Upper Extremity Scale was a method that could be used to evaluate the selective motor control of the upper extremity in children aged 3–18 years with cerebral palsy, whose content validity, reliability, construct validity, intra-, and interrater reliability have been determined (Wagner et al., 2016; Yildiz et al., 2020). The Modified Ashworth Scale was the most prevalent tool to measure the tone of specific muscles in children with cerebral palsy (Meseguer-Henarejos et al., 2018).

## Statistical analysis

SPSS 26.0 (IBM, Armonk, New York, USA) was used to carry out statistical analysis. We adopted repeated-measures analysis of variance [tDCS (active vs. sham) or Day (Day 1 vs. Day 2) × time] for all assessments of hand function. Follow-up one-way repeated-measures ANOVA was used for significant interactions and single effects for time, whereas one-way between-factor ANOVA was used for tDCS and day and corrected for multiple comparisons (Bonferroni).

The normality of data was examined by the Shapiro-Wilk test. Moreover, before running the analysis, the sphericity test for repeated measures analysis of variance was assessed by Mauchly's test; whenever assumptions were not met, Greenhouse-Geisser correction was used for violations of sphericity.

## Results

In total, 15 boys and 15 girls were recruited into this trial (mean age ± SD: 47.53 ± 11.23 months, range: 36–72 months). The ratio of damaged hemispheres on the left and right was 13:17. For MACS, 28 and 2 children were at level I and level II, respectively. For GMFCS, 26 and 4 children were at level I and level II, respectively. The characteristics of all participants were shown in Table 1.

**Table 1:** Characteristics of the participants.

**No**.	**tDCS** **order**	**Gender**	**Age** **(months)**	**HCP side**	**MACS**	**GMFCS**	**High risk factors**	**MRI**
1	AS	F	38	L	I	I	Premature birth	White matter maldevelopment
2	AS	F	37	L	I	I	NA	NA
3	AS	M	44	L	I	I	NA	NA
4	AS	F	70	R	I	I	Premature birth	NA
5	AS	F	42	R	I	II	Premature birth	NA
6	AS	F	40	R	I	II	Premature birth	NA
7	AS	M	46	L	I	I	Premature birth	Left ventricle semicovoid patch
8	AS	M	63	L	I	I	NA	NA
9	AS	F	68	R	I	I	NA	White matter maldevelopment
10	AS	F	38	L	I	I	Jaundice, hypoxia	NA
11	AS	M	51	L	I	I	Premature birth	NA
12	AS	M	53	R	I	I	NA	NA
13	AS	M	44	L	I	I	NA	NA
14	AS	M	36	R	II	I	Premature birth	NA
15	AS	M	43	R	I	I	Hypoxia	Left brain patchy lesion
16	SA	F	41	L	I	I	Premature birth	NA
17	SA	F	45	R	I	I	Meconium aspiration	NA
18	SA	M	40	R	I	I	NA	NA
19	SA	M	43	R	I	I	NA	NA
20	SA	F	61	R	I	I	NA	NA
21	SA	M	40	L	I	II	Cerebral hemorrhage	Left ventricular dilation
22	SA	F	67	R	I	I	NA	NA
23	SA	M	49	L	I	I	NA	NA
24	SA	F	41	R	I	I	NA	NA
25	SA	M	38	R	II	II	NA	NA
26	SA	M	50	R	I	I	Hypoxia	NA
27	SA	F	42	L	I	I	NA	White matter maldevelopment
28	SA	M	41	L	I	I	NA	NA
29	SA	F	72	R	I	I	NA	NA
30	SA	F	41	R	I	I	NA	Left ventricular dilation

HCP, hemiplegic cerebral palsy; No., number; M, male; F, female; A, active; S, sham; R, right; L, left; MACS, manual ability classification system; GMFCS, Gross Motor Function Classification System; NA, not available.

### Safety

No severe adverse event occurred among the 30 participants and only a few of them felt transient and slight discomfort (tingling, itching, burning sensation, dizziness, etc.). With respect to the self-report questionnaire assessing tDCS adverse events, as shown in Table 2, only a limited number of participants reported transient and slight discomfort after both active (the proportion of dizziness, burning sensation, tingling, and itching were 1/30, 1/30, 2/30, and 1/30, respectively) and sham stimulation (the proportion of tingling is 2/30). All adverse events were mild.

**Table 2:** Adverse events of participants during the study.

	**T1**		**T2**		**T3**	
**Adverse events**	**No. (Mean intensity)**		**No. (Mean intensity)**		**No. (Mean intensity)**	
	**Active**	**Sham**	**Active**	**Sham**	**Active**	**Sham**
Dizziness	1 (1)	0 (0)	0 (0)	0 (0)	0 (0)	0 (0)
Headache	0 (0)	0 (0)	0 (0)	0 (0)	0 (0)	0 (0)
Scalp pain	0 (0)	0 (0)	0 (0)	0 (0)	0 (0)	0 (0)
Burning sensation	1 (1)	0 (0)	0 (0)	0 (0)	0 (0)	0 (0)
Tingling	2 (1)	2 (1)	0 (0)	0 (0)	0 (0)	0 (0)
Drowsiness	0 (0)	0 (0)	0 (0)	0 (0)	0 (0)	0 (0)
Itching	1 (1)	0 (0)	0 (0)	0 (0)	0 (0)	0 (0)
Skin redness	0 (0)	0 (0)	0 (0)	0 (0)	0 (0)	0 (0)
Other	Absent	Absent	Absent	Absent	Absent	Absent

T1, immediately after the end of two transcranial direct current stimulation sessions (tDCS); T2, 90 min after two tDCS sessions; T3, 24 h after two tDCS sessions; No., number; Mean intensity, mean intensity range of the events (0, absent; 1, mild; 2, moderate; 3, severe).

### Improvement of unimanual function

**Figure 2:**
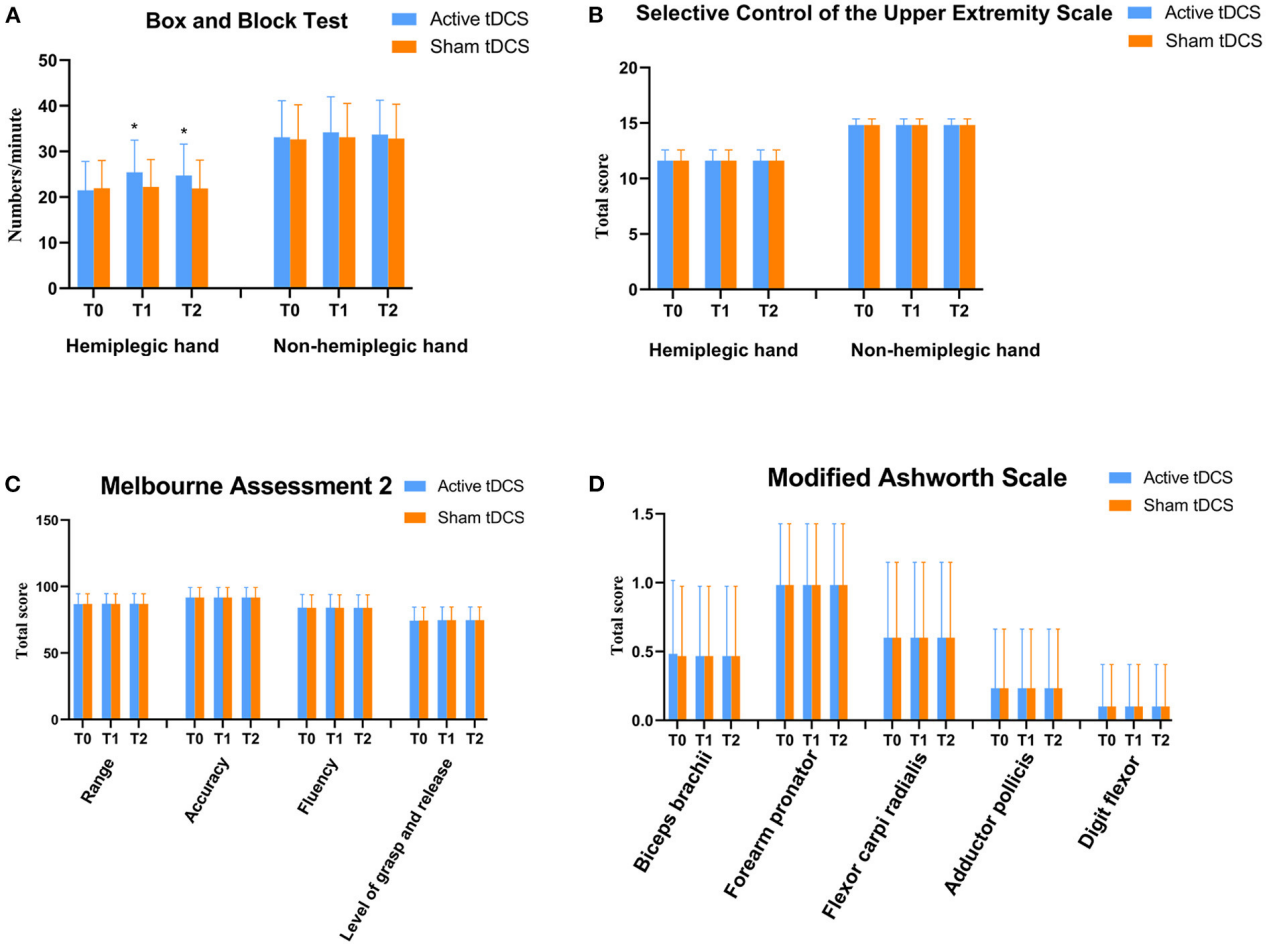
Changes of hand function in the two transcranial direct current stimulation (tDCS) sessions at T0, T1, T2. **(A)** Box and Block Test scores of both hands. **(B)** Selective Control of the Upper Extremity Scale scores of both hands. **(C)** Melbourne Assessment 2 scores of the hemiplegic hand. **(D)** Modified Ashworth Scale scores of the hemiplegic hand. T0, baseline; T1, immediately after two tDCS sessions; T2, 90 min after two tDCS sessions. *Significant difference compared to baseline (*P* < 0.05).

**Figure 3:**
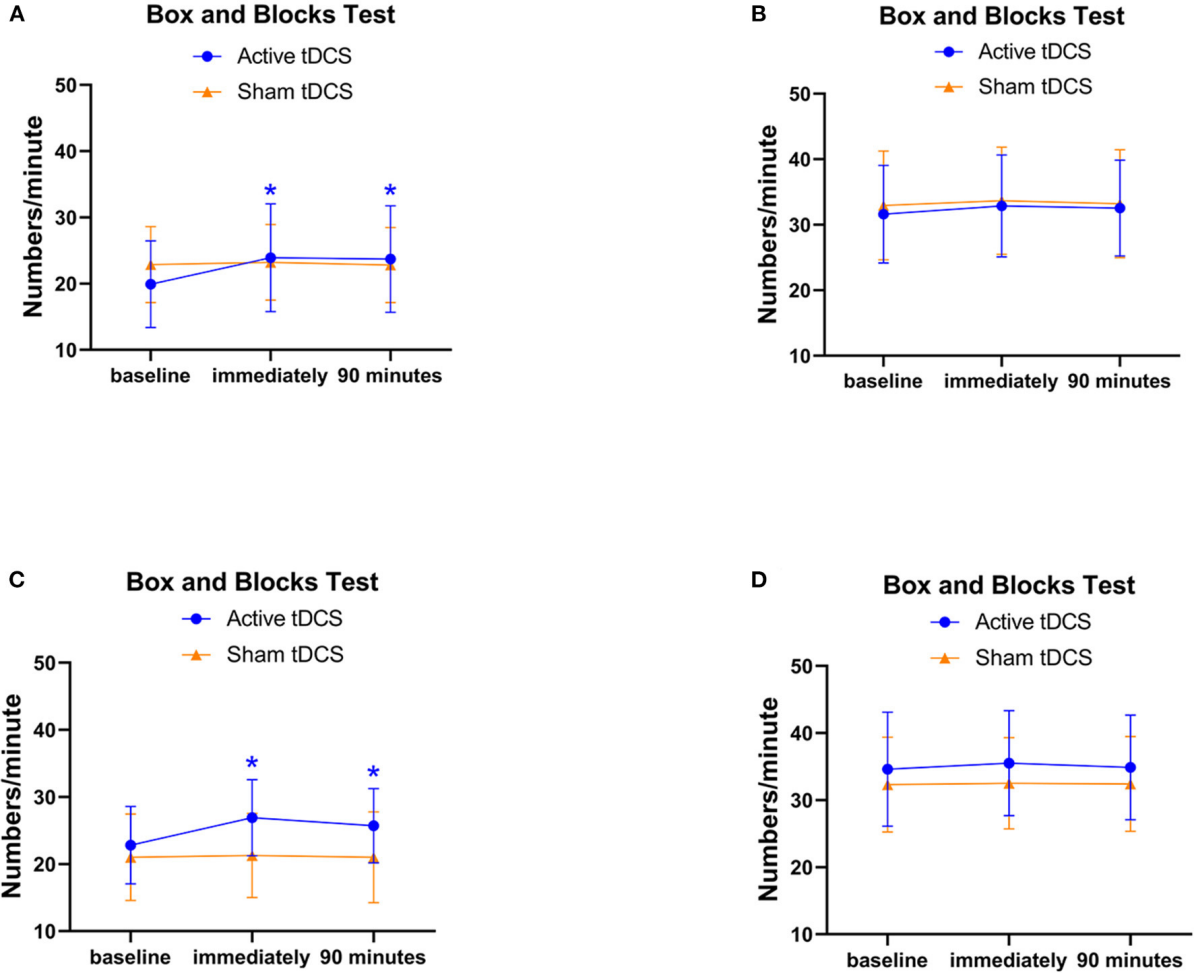
**(A)** The Box and Block Test of the affected hand in Day 1; **(B)** The Box and Block Test of the unaffected hand in Day 1; **(C)** The Box and Block Test of the affected hand in Day 2; **(D)** The Box and Block Test of the unaffected hand in Day 2; *significant difference compared to baseline (*P* < 0.05).

The affected hand of all participants performed better after accepting active tDCS at T1 and T2 compared to baseline in Box and Block Test. There was significant interaction of “tDCS × time” (*P* < 0.01) and no significant interaction of “Day × time” (*P* = 0.465). There were significant simple effect for tDCS (*P* < 0.01) and time (*P* < 0.01), whereas no significant main effect for day (*P* = 0.229). Follow-up one-way repeated-measures ANOVA was used for significant interactions and single effects for time and *post-hoc* pairwise comparison revealed significantly more at T1 (*P* < 0.01) and T2 (*P* < 0.01) compared to baseline after active tDCS and no main effect of time after sham tDCS (*P* = 0.114; Figure 2A). We also separately analyzed the Box and Block Test data of each day, the outcome showed that the affected hand performed better only after active tDCS at both Day 1 and Day 2 (T1_Day1_ vs. T0_Day1_, *P* < 0.01 and T2_Day1_ vs. T0_Day1_, *P* < 0.01; T1_Day2_ vs. T0_Day2_, *P* < 0.01; and T2_Day2_ vs. T0_Day2_, *P* < 0.01; Figures 3A,C).

In addition, we applied a pair *T*-test to assess the baseline of Box and Block Test of the affected hand for the group who received active tDCS first and significant difference was found (T0_Day1_ = 19.93 vs. T0_Day2_ = 21.00, *P* = 0.02), while there was no significant difference between T0_Day1_ and T0_Day2_ in the group who received sham tDCS first (*P* = 0.262)_._

As for the unaffected hand, there was no significant interaction of “tDCS × time” (*P* = 0.098) and no significant interaction of “day × time” (*P* = 0.244) in Box and Block Test between active and sham tDCS. There was no significant main effect of time (*P* = 0.091), Day (*P* = 0.37), and tDCS (*P* = 0.058) (Figure 2A and Table 3). The separate analysis of the Box and Block Test data showed no significant difference in T1 and T2 compared baseline in both Day 1 and Day 2 (all *P* > 0.05, Figures 3B,D). All children showed no difference in Selective Control of the Upper Extremity Scale of both hands after active or sham tDCS (Figure 2B).

**Table 3:** Comparison of Box and Block Test between the two treatment groups.

**Assessments**	**Intervention point**	**Active tDCS (*n* = 30)**	**Sham tDCS (*n* = 30)**	***P*-value**
Box and Block Test (affected hand)	T0	20.93 (5.70)	21.45 (5.57)	0.095
	T1	24.66 (5.73)	21.76 (5.49)	<0.001
	T2	24.07 (5.93)	21.34 (5.52)	<0.001
Box and Block Test (unaffected hand)	T0	33.10 (8.01)	32.63 (7.60)	0.098*
	T1	34.20 (7.79)	33.10 (7.41)	
	T2	33.70 (7.53)	32.80 (7.57)	

P-value represents between-group differences. Data shown are means (SD). tDCS, transcrainal direct current stimulation; T0, baseline; T1, immediately after the end of two tDCS sessions; T2, 90 min after the two tDCS sessions. ^*^Interaction “tDCS × time” showed no significant difference in this index.

### Affected upper extremity performance

There was no difference for the four sub-scales of Melbourne Assessment 2 (range of motion, level of grasp and release, accuracy, and fluency; Figure 2C). As for muscular tone, nearly all children showed no difference in the outcome of Modified Ashworth Scale (biceps brachii, forearm pronator, flexor carpi radialis, adductor pollicis, and digiti flexor; Figure 2D).

## Discussion

This study aimed to investigate the safety as well as the immediate and short-term effects of a single anodal tDCS (1.5 mA) over M1 on the upper limb function for preschool children. No serious adverse event occurred during this study. The outcomes also showed that a single anodal tDCS (1.5 mA, 20 min) over the affected M1 improved dexterity of the affected hand for preschool children with HCP. These results complemented the existing evidence on the safety and effects of tDCS (1.5 mA, 20 min) in preschool children with HCP.

Consistent with previous studies (Ciechanski and Kirton, 2017; Gillick et al., 2018), no serious adverse event occurred among all children during and after our experiment. Only a few of participants felt transient and slight discomfort (tingling, itching, burning sensation, dizziness, etc.), which occurred in both active tDCS stimulation and sham tDCS stimulation. And these adverse effects also occurred in similar investigations with higher incidence (Mutlu et al., 2008; Inguaggiato et al., 2019). The recorded lower frequency of adverse events in our study might attribute to the parameters of tDCS and the poorer ability to describe uncomfortable feelings for preschool children. When adverse events occurred, we would stop the intervention as soon as possible and activated code blue if needed and we had a professional medical team to track every participant all the way. Our study provided evidence for safety of a single anodal tDCS over M1 at used parameter (1.5 mA, 20 min) for preschool children with HCP.

A previous study indicated that younger children obtained lower scores than older children with HCP in Box and Block Test which means poorer hand dexterity (Jongbloed-Pereboom et al., 2013). According to a research about the reliability and responsiveness of the Box and Block Test for children with cerebral palsy, the clinical significant difference for the Box and Block Test was 1.9 (blocks) on the more affected hand (Araneda et al., 2019). The results of this study showed a change in the Box and Block Test of the affected hand at T1 (3.73 blocks) and T2 (3.14 blocks) compared to T0, which indicated that preschool children with HCP performed better in Box and Block Test of the affected hand after a single anodal tDCS (1.5 mA, 20 min) over the affected M1; improvement was found immediately after stimulation and lasted for at least 90 min. Meanwhile, the significant difference of T0_Day1_ and T0_Day2_ for the group who received a single active tDCS firstly indicated that this positive effect might last over 24 h, which differed from a similar study (Inguaggiato et al., 2019). According to a study about the impact of age on tDCS (Saldanha et al., 2020), we considered that the difference of age in the focused population (preschool children vs. individuals aged 10–28 years old) might account for this inconsistent result. The plasticity-dependent effects induced by tDCS indicated that the brain of preschool children featured with developing cortical and corticospinal excitability might benefit more from this tool than school-aged children and adult individuals with HCP. In short, this improvement was temporary rather than long-term, which was consistent with previous study. For example, a single session of anodal tDCS over the primary motor cortex of the hemisphere ipsilateral to the brain lesion led to momentary motor improvements in both upper limbs of the children with spastic hemiparetic CP in a study (Moura et al., 2017). Another study also indicated that a single anodal tDCS temporarily improved hand dexterity skills for patients in the subacute phase of stroke (Fusco et al., 2014). Contrast to the hemiplegic hand, the dexterity of non-hemiplegic one was not weaken by stimulation, which was in line with a previous study (Inguaggiato et al., 2019). Although there was no significant difference, we noticed that some participants got higher scores at the Box and Block Test of the non-hemiplegic hand after active tDCS rather than sham tDCS. Because of the interhemispheric competition and inhibition, the loss of inhibition over the unaffected hemisphere from the affected hemisphere caused the increased excitability of the unaffected hemisphere for individuals with HCP. Based on that anodal tDCS might improve hand dexterity by upregulating the excitability of the lesioned motor cortex, the different pattern of interhemispheric competition, and inhibition might contribute to the outcome of non-paretic hand. According to previous study, greater hemisphere excitation was associated with greater gains in motor function (Cunningham et al., 2015).

With regard to Melbourne Assessment 2, Selective Control of the Upper Extremity Scale, Modified Ashworth Scale, no positive effect emerged after a single anodal tDCS, which might be due to the following reasons. For one thing, the Box and Block Test was to test the hand dexterity featuring advantages of simplicity of operator, high reliability, and responsiveness (Araneda et al., 2019). The Melbourne Assessment 2 tested the affected hand function and motor quality and the Selective Control of the Upper Extremity Scale tested selective motor control of the upper extremity but their responsiveness to determine whether it could assess therapy-induced improvements remains to be determined (Elvrum et al., 2016; Lieber et al., 2021). The Modified Ashworth Scale mainly tested the muscle tone. The Box and Block Test was sensitive to changes produced by a single anodal tDCS in hands due to its high reliability and responsiveness. Conversely, the Melbourne Assessment 2 and the Selective Control of the Upper Extremity Scale might be less sensitive. Secondly, for the Modified Ashworth Scale, a single anodal tDCS might produce no effect on muscle tone, which was consistent with previous study (Comino-Suárez et al., 2021).

Limitations of the present study were that the washout period of 24 h was not long, which might lead to significant difference on T0_Day1_ and T0_Day2_ for the group who received active tDCS first. Also, this study was not double blind. It was indicated that the wash-out time should be longer in future similar study. At the same time, relevant clinical information on brain lesions and injuries for the part of participants was incomplete. Lastly, according to the available MRI information, there were variety of findings related to white matter injuries or malformations, but the anodal tDCS only applied to presumed M1, which might neglect the relationship of lesions/injuries and stimulation area.

Studies with larger samples about the optimal dose, duration, and treatment cycle of tDCS for preschool children with HCP are warranted. On the other hand, there are some views that the timing, severity of brain lesion and the individual corticospinal tracts projections in HCP might exert influence on tDCS efficacy (Gillick et al., 2018). Further researches are needed to focus on these points, thus providing more help for applying tDCS into neurodevelopmental rehabilitation in pediatric population.

## Conclusion

A single application of anodal tDCS (1.5 mA, 20 min) over M1 safely and tolerably improved the affected hand dexterity for preschool children with HCP.

## Data availability statement

The original contributions presented in the study were included in the article/supplementary material, further inquiries can be directed to the corresponding author/s.

## Ethics statement

The studies involving human participants were reviewed and approved by Guangzhou Women and Children's Medical Center Research Ethics Committee. Written informed consent to participate in this study was provided by the participants' legal guardian/next of kin.

## Author contributions

KX conceived this work, contributed to study design, project management, and fund procurement. WH and YH wrote this manuscript and performed data collection and analysis. LH and LL generated the figures and tables. PZ contributed to guidance on English writing. HZ, ZC, YX, JZ, and WW carried out literature search. HQ and XW contributed to participant recruitment. KX and HT revised the manuscript. All authors have read and approved the content of the manuscript.

## Funding

This work was supported by the National Natural Science Foundation of China (81902309, 81672253), the Natural Science Foundation of Guangdong Province (2019A1515010420, 2021A1515011303, 2021A1515012543), the Basic and Applied Basic Foundation of Guangdong Province (2020A1515110435), and funds from the Guangzhou Municipal Science and Technology Project (202102010205, 202102020581) and the Featured Clinical Technique of Guangzhou (2019TS55). The funders played no role in the design, conduct, or reporting of this study.

## Conflict of interest

The authors declare that the research was conducted in the absence of any commercial or financial relationships that could be construed as a potential conflict of interest.

## Publisher's note

All claims expressed in this article are solely those of the authors and do not necessarily represent those of their affiliated organizations, or those of the publisher, the editors and the reviewers. Any product that may be evaluated in this article, or claim that may be made by its manufacturer, is not guaranteed or endorsed by the publisher.
